# Physical Activity during COVID-19 Lockdown: Data from an Italian Survey

**DOI:** 10.3390/healthcare9050513

**Published:** 2021-04-28

**Authors:** Matteo Guidetti, Alberto Averna, Greta Castellini, Michelangelo Dini, Daniela Marino, Tommaso Bocci, Roberta Ferrucci, Alberto Priori

**Affiliations:** 1 Aldo Ravelli Research Center for Neurotechnology and Experimental Neurotherapeutics, Department of Health Sciences, University of Milan, 20142 Milan, Italy; matteo.guidetti@unimi.it (M.G.); alberto.averna@unimi.it (A.A.); michelangelo.dini@unimi.it (M.D.); tommaso.bocci@unimi.it (T.B.); roberta.ferrucci@unimi.it (R.F.); 2 Department of Electronics, Information and Bioengineering, Politecnico di Milano, 20133 Milan, Italy; 3 Faculty of Agricultural, Food and Environmental Sciences, Catholic University of the Sacred Heart, 26100 Cremona, Italy; greta.castellini@unicatt.it; 4 ASST Santi Paolo e Carlo, 20142 Milan, Italy; spazioat.blogspot@gmail.com

**Keywords:** COVID-19, mental health, physical health, quality of life, IPAQ, PGWB-S

## Abstract

The COVID-19 pandemic has forced governments to impose quarantines and lockdowns as containment strategy, raising concerns about mental health and low level of physical activity performed by quarantined populations. In this study, we assess the level of physical activity and psychological wellbeing in a sample of the Italian population during lockdown through an online format of International Physical Activity Questionnaire (IPAQ) and Psychological General Well-Being index—Short version (PGWB-S). Of 317 adult responders considered, most were female (61.2%), young adults (52.4%), living in little-to-medium size cities (80.1%) and with high-level education (62.8%). Most of our sample performed physical activity mostly during leisure time and domestic activities, and 60.9% were highly active. No interactions were found between physical activity and the demographic characteristics considered. Subjects performing high level of physical activity felt more energetic and vital than those with moderate (*p* < 0.0001) and low levels (*p* < 0.0001) of physical activity. Our participants performed enough activity to satisfy the WHO Guidelines, mainly due to domestic activity and activity performed during leisure time, with an overall moderately positive psychological reaction to lockdown.

## 1. Introduction

In 2020, the diffusion of a novel virus (SARS-COV 2) causing severe respiratory syndrome (COVID-19) resulted in a global health crisis [1] and put great pressure on health systems [2]. Several governments decided for social distancing, quarantine and temporary nationwide lockdown as containment strategies [3]. Such interventions impose changes of personal habits, with great psychological stress [4]. A recent systematic review has suggested that almost one out of every five people undergoing restrictive measures is at risk of developing clinically significant psychological distress [5]. Similarly, social restrictions affect the possibility of physical movement, possibly turning the forced reduction of mobility and physical activity (PA) into established sedentary behaviours [6]. According to the World Health Organization (WHO) [7], sedentary behaviour is defined as “any waking behaviour characterized by an energy expenditure of 1.5 Metabolic Equivalents of Task (METs) or lower while sitting, reclining or lying“, while physical activity is “any bodily movement produced by skeletal muscles that requires energy expenditure”. Therefore, great concerns have been raised about the risk of negative consequences on mental and physical health of quarantined people [4], suggesting that people would not be able to satisfy minimal levels of PA to maintain health [8]. Indeed, the WHO Guidelines for PA [7] recommend carrying out at least 450–900 METs-min/week of PA (i.e., at least 150–300 min of moderate-intensity aerobic PA, or at least 75–150 min of vigorous-intensity aerobic PA, or an equivalent combination of moderate- and vigorous-intensity activity throughout the week) to stay healthy. Energy expenditure is commonly expressed as MET, a unit of measure which refers to the rate of energy expenditure while sitting at rest [9]. Moderate-intensity PA implies approximately 3–6 METs, while vigorous implies >6 METs.

Italy was the first European country facing the COVID-19 emergency at the end of February 2020, with the Italian population formally quarantined on 11 March 2020 [10]. Movement outside of the house was totally forbidden, except for proven needs or exercising individually within few meters from home. Such strict conditions were gradually suspended on 4 May 2020.

In this study, we report data collected through an online survey from 28 April 2020 to 17 May 2020 (i.e., during the lockdown and the first reopening) from a sample of the Italian population to assess the level of PA and psychological well-being during the lockdown.

## 2. Materials and Methods

We designed a survey for online completion during the SARS-COV 2 outbreak. It comprised 35 questions, assessing demographic factors (sex, age, highest level of educational qualification, size of the city where the lockdown was spent), PA levels and psychological aspects.

PA was assessed through the Italian long version of the International Physical Activity Questionnaire (IPAQ) [11]. This tool provides a quantitative score (in METs-min/week) of PA performed in the 7 days prior to questionnaire completion for 5 activity domains (job-related PA; transportation PA; housework, house maintenance and caring for family; recreation, sport, and leisure-time PA; time spent sitting). For the purpose of this article, we excluded job-related PA and time spent sitting. Following the IPAQ guidelines [12], which take into account the frequency, intensity, duration, and type of PA performed, participants were classified in 3 levels: “low PA”, “moderate PA” and “high PA”.

Psychological conditions were investigated through the Psychological General Well-Being index—Short version (PGWB-S) [13], a validated questionnaire assessing subjective perception of well-being in the 28 days prior to questionnaire completion. It consists of 6 subitems that evaluate different aspects of psychological well-being (anxiety, vitality-energy, depressed mood, self-control, positive well-being, vitality-tiredness). Each subitem is rated on a 6-point Likert scale (range = 0–5; maximum global score = 30). Responses are scored so that higher total PGWB-S scores indicate higher psychological wellbeing [13].

We used a Computer Assisted Web Interviewing (CAWI)) methodology, with the survey questions available on an online Google Form from 28 April 2020 to 17 May 2020. 330 participants took part, but only 321 questionnaires were completed (response rate: 96.06%). The convenience sample of participants was reached through social media and instant messaging platforms. As we aimed to reach the general population, the only inclusion criterion was the age of majority (>18 years). After collection, data were exported for analysis. Data gathering and analysis occurred guaranteeing the compliance with current legislation on the collection and processing of personal data. All subjects gave online, written informed consents before the participation, and the study protocol followed the Declaration of Helsinki.

Descriptive statistics were used to calculate categorical variables: age (18–30; 31–65; >65), sex (male; female), education (5–8 years; 8–13 years; 13–17 years; >17 years) and city size (<10 thousand residents; 10–100 thousand residents; >100 thousand residents). Frequency analysis was then used to assess the level of PA based on categorical variables (age, sex, education, city size).

χ2 test was used to assess associations between the levels of PA (low, moderate, high) and age, sex, education, and city size. Besides these, IPAQ-total score and PA domains scores (transportation, leisure time, domestic activities) were considered as METs-min/week and considered as non-normally distributed [12]. Independent-samples Kruskal–Wallis analysis of variance and boxplots of subitems normalized to total IPAQ score were used to explore the extent to which each activity domain contributed to the total score. Pearson correlation explored the relationship between IPAQ-total score and PGWB-S total score.

One-way ANOVA was used to assess whether PGWB-S total score varied across the levels of PA. Similarly, Independent-samples Kruskal–Wallis analysis of variance was used to explore differences in each PGWB-S subitem across the levels of PA. Statistical analysis was performed using SPSS ver. 23 (IBM, Chicago, IL, USA).

## 3. Results

### 3.1. Descriptive Characteristics

Among the 321 valid responses, we considered for analysis only the 317 which were completed before 4 May 2020, i.e., when the restrictions were more stringent. Missing data have been excluded from the analysis. Most of the subjects were young (52.4%), female (61.2%), and with tertiary level education (62.8%). The majority came from little-to-medium size cities (80.1%) (see Table 1).

### 3.2. IPAQ

Descriptively, the majority of the sample reported high levels of PA (60.9%). Higher level of PA was reached by young subjects (66.3%) and those with 13–17 years of education (64.4%). However, the χ2 tests revealed no significant interactions between PA levels and demographic variables.

**Figure 1:**
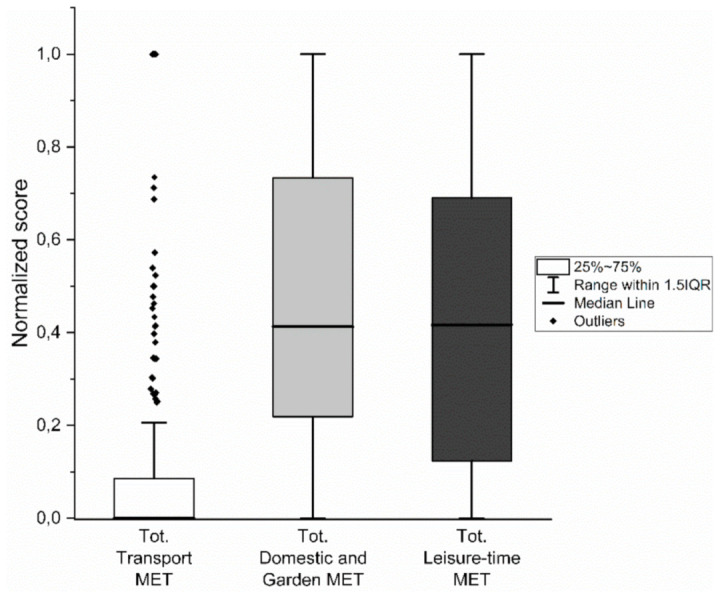
Box plots representing normalized distribution of IPAQ domains.

Our sample was physically active mostly during domestic and garden activities (METs-min/week, median—IQR = 878–2051.5) and during leisure time (1070–1902.5) (see Table 2). Independent-Samples Kruskal–Wallis analysis pointed out a significant difference between METs-min/week performed in each domain (χ2 [2] = 284.9, *p* < 0.0001). Dunn’s pairwise tests revealed that PA performed during transportation was lower than PA performed during domestic activities (*p* < 0.0001) and during leisure time (*p* < 0.0001). No difference was found between domestic activities and leisure time (*p* = 1.000). To examine the extent to which each IPAQ domain affected the total score, a graphical representation of the domains normalized to the total IPAQ score is presented (see Figure 1).

### 3.3. PGWB-S

Mean PGWB-S global score was 19.54 ± 4.65. Among the subitems explored, the highest score was observed in the subitem assessing anxiety (3.7 ± 1.21), which indicates that subjects reported low levels of anxiety. Lower scores were recorded in positive well-being (2.59 ± 1.09), which indicates that subjects experienced fewer positive thoughts (see Table 3).

**Figure 2:**
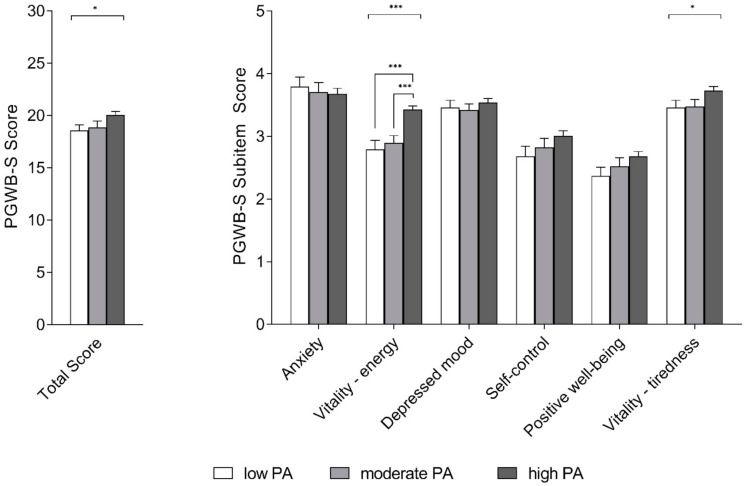
Psychological well-being in participants across the levels of PA (low, moderate, high). Left: PGWB-S total score; Right: PGWB-S subitems. Data are presented as mean score with standard error. Asterisks denote statistical significance (* = *p* < 0.05; ** = *p* < 0.01; *** = *p* < 0.001).

PGWB-S global scores were found to be normally distributed (Kolmogorov–Smirnov test: *p* > 0.05). One-way ANOVA revealed significative differences for PGWB-S global score across the levels of PA (high, moderate, low) (F (2.314) = 3.336, *p* = 0.037). However, Bonferroni post-hoc tests could not show any further difference (see Table 3 and Figure 2).

Independent-samples Kruskal–Wallis analysis to assess difference of PGWB-S subitems scores across the levels of PA showed significative differences for the vitality-energy subitem (χ2 [2] = 26.87, *p* < 0.0001). Pairwise comparisons revealed that vitality-energy score was significantly higher in the “high PA” group, compared to the “low PA” group (*p* < 0.0001) and “moderate PA” group (*p* < 0.0001). No differences were found between “low PA” and “moderate PA” (*p* = 1.000) (see Table 3 and Figure 2).

Independent-Samples Kruskal–Wallis analysis also revealed significant differences for the vitality–tiredness subitem across the levels of PA (χ2 [2] = 6.37, *p* = 0.04). However, pairwise comparisons could not show any further differences (see Table 3 and Figure 2).

Moreover, IPAQ-total score showed a weak positive correlation with PGWB-S total score (r = 0.17, *p* < 0.0001).

## 4. Discussion

In this study, we explored the level of PA of a sample of 317 Italian citizens through an online survey during the first wave of the COVID-19 pandemic (April–May 2020). According to literature [14], lockdown measures induce increased risk of inactivity, with possible health implications. Results from available survey studies on the effect of lockdown on physical activity performed by the population reveal a complex situation, with PA affected by numerous factors during public health emergencies [15]. Generally, results tend to confirm the foretold risks of physical inactivity. Studies performed on different European [16,17,18,19], Australian [20] and Chinese populations [15] reported a general decrease of quantity and type of PA performed.

Differently, our results report that more than 80% of our sample performed moderate to high level of physical activity in the 7 days prior to questionnaire completion. Such results were obtained because, in the context of social restrictions and limitations of freedom, our participants were physically active mostly during leisure time and taking care of the garden/household, as recommended [21]. Indeed, this allowed them to have a sufficiently active lifestyle to stay healthy, according to the WHO Guidelines for Physical Activity [7]. Interestingly, such level of PA has been found to reduce the prevalence of COVID-19-related hospitalization and suggested as a strategy to reduce the impact of SARS-CoV-2 infection, especially in the elderly [22]. These results might be related to the ability of PA to improve immune system activity and efficiency [23] and reduce inflammation [24]. Reducing COVID-19 hospitalization rates should become a priority, as recent evidence suggest that patients hospitalized for COVID-19 tend to exhibit lasting cognitive impairment in the months following hospital discharge [25].

Remarkably, some findings disclosed that those who were poorly active [18,26] or exercised only occasionally [27] before COVID-19 lockdown, increased weekly energy expenditure and exercise performing. Indeed, in the study from Maugeri et al. [18], participants defined as “low active” before lockdown significantly increased total weekly physical activity energy expenditure during lockdown; the authors explained such a result with the greater amount of housework activities carried out by these participants forced to stay at home. Indeed, similarly to sporting activities such as running or playing soccer, exercises performed at home, such as moving heavy loads or climbing stairs are considered to be vigorous activity [28]. Moreover, Bourdas et al. [16] reported that leisure time activities increased by almost one fifth during lockdown, although such changes could not counteract the large reduction of daily occupational activities (−52.9%), activities related to transportation (−41.1%), and sporting activities (−23.9%). It should also be noted that quarantined people have more free time, which they could use to perform those physical activities that have been reported by previous studies (for example, exercising in leisure time, doing housework).

Several studies reported differences in reductions of PA between males and females [16,18,19]. In detail, males were found to experience a reduction of METs-min/week in greater extent than females. Such results might have a psychosocial reason [16]. Indeed, social distancing policies and sports restrictions might have dissuaded males more than females, since the literature reports that males seem to find a motivation to practice sports or PA to have fun, to improve their performances, and to spend time with friends [16]. Differently, females stay active more to control weight and to improve their physical appearance [16].

As for age, results are heterogeneous. Giustino et al. [19] found an inverse relationship between PA level and age, with greater reduction of PA in participants up to 55 years old (i.e., young, young adults and adults). Similarly, Qin et al. [15] showed that young adults (20–34 years) had a higher prevalence of insufficient PA and those over 55 years old had a lower prevalence. However, López-Sánchez et al. [17] found that people between 45 and 54 did not significantly decrease PA in lockdown. Although without reaching statistical significance, our sample showed a trend in which males and younger subjects reached the highest rates of physical activities.

Lastly, the analysis of the psychological conditions revealed that our sample did not report lower psychological wellbeing, compared to results observed in the Italian normative study [13] and other studies which have used the PGWB-S [29]. Such a result could seem to be in contrast with the available literature also related to the Italian COVID-19 outbreak [30], which describes the significant negative impact of lockdown on psychological health, detectable in the shape of post-traumatic stress symptoms, confusion, anger [4], and changes in sexuality [31]. However, one should consider the context in which our survey was performed, i.e., the last week of lockdown. In this period, the infection slowdown was ever more prominent, the health services crisis had begun to ease, and people were already aware about the forthcoming reopening, with summertime drawing near. Moreover, our sample exhibited a moderate-to-high level of activity, and this might have had a beneficial effect on psychological health, as extensively reported [32]. This hypothesis is also supported by a positive, if weak, correlation between level of PA and psychological wellbeing. Additionally, vitality–energy was found to be significantly higher in participants performing higher levels of PA. A previous similar study [18], performed on the Italian population before and after lockdown, widely confirmed that the reduction of physical activity levels is related to a worse status of psychological well-being. Similar results were reached by a Chinese study [15]. Indeed, regular exercise lowers depressive and anxiety symptoms through the modulation of physiological processes involved in stress reactivity, anxiety, mood and emotional responses [33].

Although this research adds further knowledge to such an important topic, it has some limitations. Our sample was quite small and not balanced, and the PA considered in this study was the one performed during the 7 days prior to the questionnaire, which could reduce the generalizability of the results to the behaviour during the whole lockdown, and to the general population. Specifically, older subjects (>65 years old) were underrepresented in the study, and minors (and more importantly, adolescents) were not considered. This affects the possibility to draw conclusions for such populations. Furthermore, the lack of pre-lockdown data did not allow us to assess the actual effect of the restrictions on PA of participants, while more data would be needed to clearly understand the change in type of PA that took place during the lockdown.

## 5. Conclusions

In conclusion, the results of this online survey underline that our sample was physically active during social restrictions, performing high grades of activity in domestic and garden activities, as well as during leisure time. This might suggest that levels of physical activity sufficient to stay healthy are obtainable by engaging in such daily activities; otherwise, home-based exercise and sport training might be useful to maintain or improve physical performances. Mass media and health professionals should raise awareness about this issue, given the importance of moderate PA also during the ongoing COVID-19 threat [22]. However, many factors are involved in determining the level of physical activity one performs during a public health emergency. Such phenomena need to be further investigated, given the fundamental importance that PA has for a healthy lifestyle.

## Figures and Tables

**Table 1:** Demographic characteristics of the sample.

Variable	Category	Subjects
*n*		317
Age-*n* (%)	18–30 years	166 (52.4%)
	31–65 years	132 (41.6%)
	>65 years	19 (6%)
Sex-*n* (%)	Male	123 (38.8%)
	Female	194 (61.2%)
Education-*n* (%)	5–8 years	11 (3.5%)
	8–13 years	17 (5.4%)
	13–17 years	90 (28.4%)
	>17 years	199 (62.8%)
City size-*n* (%)	<10 thousand residents	128 (40.4%)
	10–100 thousand residents	126 (39.7%)
	>100 thousand residents	58 (18.3%)
	Missing	5 (1.6%)

**Table 2:** International Physical Activity Questionnaire (IPAQ) results. The table shows the demographic characteristic of the sample according to the level of PA. METs-min/week for IPAQ domains are reported for the whole sample and according to demographic variables.

		IPAQ-Levels ^1^			IPAQ-Domains ^2^			
		Low Physical Activity	Moderate Physical Activity	High Physical Activity	Active Transportation	Domestic and Garden Activity	Leisure Time	Total Score
Total (*n* = 317)		55 (17.4%)	69 (21.8%)	193 (60.9%)	0 (198)	878 (2051.5)	1070 (1902.5)	2408 (3460.5)
Age	18–30 years	27 (16.3%)	29 (17.5%)	110 (66.3%)	0 (148)	810 (1552,5)	1260 (1829.3)	2547 (2735.3)
	31–65 years	26 (19.7%)	33 (25%)	73 (55.3%)	0 (248)	986 (2499)	791 (1901.5)	2233 (4090.5)
	>65 years	2 (10.5%)	7 (36.8%)	10 (52.6%)	164 (370.5)	1901 (6603.8)	1040 (1211.8)	3695 (7520.8)
Sex	male	16 (13%)	28 (22.8%)	79 (64.2%)	50 (270)	810 (2093)	1260 (1800)	2470 (3573)
	female	39 (20.1%)	41 (21.1%)	114 (58.8%)	0 (148)	945 (2036)	900 (1909.5)	2397 (3362)
Education	5–8 years	4 (36.4%)	1 (9.1%)	6 (54.5%)	99 (297)	1185 (1755)	0 (1040)	2097 (4654)
	8–13 years	5 (29.4%)	3 (17.6%)	9 (52.9%)	0 (198)	900 (2186)	300 (2123)	2150 (3867)
	13–17 years	8 (8.9%)	24 (26.7%)	58 (64.4%)	0 (283.5)	967.5 (2086.8)	1240 (1985.5)	2800.5 (3617.8)
	>17 years	38 (19.1%)	41 (20.6%)	120 (60.3%)	0 (156)	859 (1946.3)	1040 (1841)	2404.5 (3392.3)
City size	<10,000 residents	21 (16.4%)	26 (20.3%)	81 (63.3%)	0 (148)	1312.5 (2715)	1040 (2233.5)	2886.5 (4370.5)
	10–100 thousand residents	22 (17.5%)	28 (22.2%)	76 (60.3%)	0 (156)	761 (1426.5)	1080 (1616)	2303 (2464.3)
	>100 thousand residents	11 (19%)	12 (20.7%)	35 (60.3%)	74.5 (332.5)	731.5 (1316.3)	1110 (1670)	2182.5 (3292.3)

^1^*n* (%); ^2^ median (IQR) of METs-min/week.

**Table 3:** Psychological General Well-Being index—Short version (PGWB-S) results. The table shows PGWB-S total score and subitems for the whole sample, as well as according to PA levels.

PGWB-S	Total	Activity Levels			*p* Value
		Low ^1^	Moderate ^1^	High ^1^	
anxiety	3.7 ± 1.21	3.80 ± 1.11	3.71 ± 1.24	3.68 ± 1.23	0.941
vitality-energy	3.21 ± 0.91	2.80 ± 1.05	2.90 ± 0.96	3.43 ± 0.78	**<0.0001**
depressed mood	3.5 ± 0.84	3.46 ± 0.84	3.42 ± 0.83	3.54 ± 0.85	0.534
self-control	2.91 ± 1.21	2.69 ± 1.18	2.83 ± 1.18	3.01 ± 1.22	0.173
positive well-being	2.59 ± 1.09	2.37 ± 1	2.52 ± 1.13	2.68 ± 1.10	0.121
vitality-tiredness	3.63 ± 0.89	3.46 ± 0.84	3.48 ± 0.92	3.73 ± 0.88	**0.041**
PGWB-S total score	19.54 ± 4.65	18.57 ± 4.08	18.86 ± 4.99	20.07 ± 4.63	**0.037**

^1^ mean ± standard deviation; *p* values reflects significance of ANOVA for PGWB-S total score and Kruskal–Wallis analysis for PGWB-S subitems.

## Data Availability

Data not provided in the article must be shared at the request of other investigators for purposes of replicating procedures and results.
